# Repair of Inguinal Bladder Hernias Concomitant with Localized Prostate Cancer: A Case Report and Review of the Literature

**DOI:** 10.1155/2020/8877694

**Published:** 2020-12-11

**Authors:** Kenichi Hata, Kazuhiro Takahashi, Takahiro Kimura, Shin Egawa

**Affiliations:** ^1^Department of Urology, Atsugi City Hospital, 1-16-36, Mizuhiki, Atsugi City, Kanagawa-ken 243-8588, Japan; ^2^Department of Urology, Jikei University School of Medicine, 3-25-8, Nishi-Shinbashi, Minato-ku, Tokyo 105-8461, Japan

## Abstract

This study reports two rare cases of inguinal bladder hernias accompanied by localized prostate cancers. They were treated with simultaneous repair of inguinal bladder hernias and open retropubic radical prostatectomy. Additionally, we performed a literature review on previous inguinal bladder hernias case reports. In this present study, the first patient was a 64-year-old man histopathologically diagnosed with prostate cancer; computed tomography for staging of prostate cancer revealed a “Pelvic Mickey Mouse Sign.” The second patient was a 75-year-old man with right inguinal swelling that gradually increased in size for 30 years. He was referred to our department due to nocturia and urge incontinence. His prostate-specific antigen level was 4.17 ng/mL, and a transrectal prostate biopsy revealed prostate cancer. Preoperative imaging studies revealed a right hernia wherein most of the bladder slid beyond the inguinal channel filling the scrotum. Both patients underwent the Lichtenstein technique for inguinal bladder hernias simultaneously with retropubic radical prostatectomy using separate surgical incisions to avoid urinary contamination of the mesh. In our comprehensive review of patients who underwent inguinal bladder hernias surgical repair, there were 51 cases (50 males and 1 female). The mean patient age was 60.6 ± 12.3 years. Five cases demonstrating concomitant prostate cancer were observed. This present case report is the first to describe two patients who underwent surgeries for the simultaneous repair of inguinal bladder hernias and retropubic radical prostatectomy with separate surgical incisions. Supposedly, this simultaneous approach is suitable for concomitant inguinal bladder hernias and prostate cancer treatment.

## 1. Introduction

Inguinal bladder hernias (IBHs) have been reported for more than 100 years [1]. They occur in approximately 1–4% of all cases of inguinal hernias [2]. The frequency of occurrence has hardly changed over the years because of the difficulty in establishing diagnosis prior to hernioplasty. Less than 7% of IBHs are diagnosed preoperatively, and 16% of IBH cases are diagnosed postoperatively because of urinary leakage from wounds or sepsis owing to inadvertent bladder injuries during operation [3]. It is important to establish diagnosis before any surgical procedure to avoid life-threatening complications. Bisharat et al. recommended resection of the bladder if a bladder diverticulum, an incidental bladder tumor, or bladder necrosis is present in hernias or hernias neck is less than 0.5 cm in diameter [4]. Therefore, not only is preoperative diagnosis important but also operative strategy is. Notably, this importance becomes more pertinent when genitourinary malignant diseases coexist. Several IBH patients with genitourinary malignancies, such as bladder cancer and prostate cancer (PCa), have been reported [5–8]. However, patients who underwent a simultaneous repair of IBHs with radical retropubic prostatectomy (RRP) have not been previously reported. This study presents two patients who underwent repair of IBHs simultaneously with RRP. Additionally, we performed a literature review on IBH surgical procedures.

## 2. Case Presentation

This study was conducted in accordance with the Declaration of Helsinki (1964). All patients provided written informed consent and the study was approved by the responsible Ethical Committee.

### 2.1. Case 1

**Figure 1:**
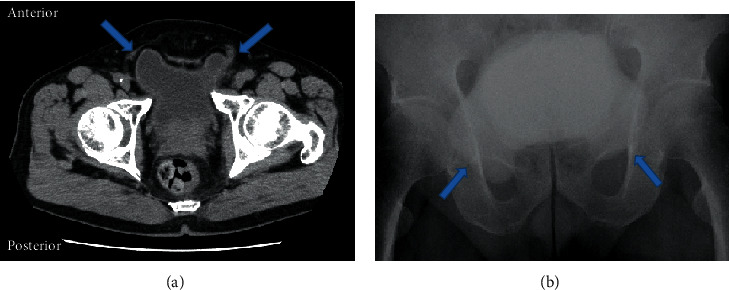
The images of case 1 showing the bladder in the right inguinal hernia. (a) Axial CT sections illustrate a small portion of the urinary bladder protruding into the bilateral inguinal canal with a “Mickey mouse”-like appearance. (b) Cystography shows the inverse “Mickey mouse”.

**Figure 2:**
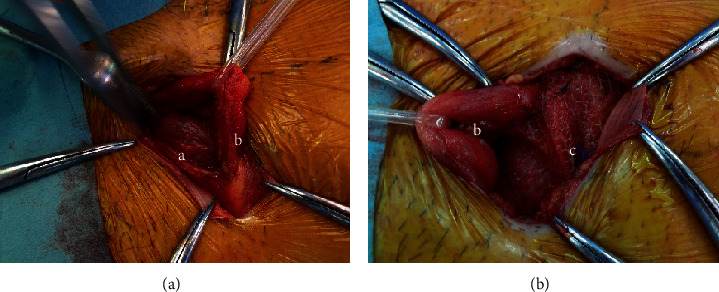
Intraoperative appearance of the herniated bladder in case 1. (a) Herniated bladder. (b) Spermatic cord. (c) Polypropylene mesh.

A 64-year-old man with prostate-specific antigen (PSA) of 25.29 ng/mL was referred to the Department of Urology, Atsugi City Hospital, Japan. He had a medical history of hypertension, diabetes mellitus, hypercholesterolemia, and epilepsy because of sequelae after a traffic accident. The patient reported no urinary symptoms or previous episodes of urinary retention. Physical examination was unremarkable. His body mass index was 26.01 kg/m^2^. He underwent a transrectal prostate needle biopsy and was histopathologically diagnosed with PCa; the Gleason score was 4 + 5. Computed tomography (CT) for PCa staging incidentally revealed bilateral inguinal hernias with the bladder protruding anteroinferiorly on both sides without metastasis (Figure 1(a)). This yielded the appearance of a “Pelvic Mickey Mouse Sign,” which Sagar et al. and Lichtenstein and Shulman described as an IBH phenomenon [9, 10]. Cystography showed the same “Mickey mouse” phenomenon with the bladder protruding into bilateral hernias mimicking the ears and the rest of the bladder resembling the face (Figure 1(b)) [9]. He underwent simultaneous RRP and IBH repair with the Lichtenstein technique (PerFix Light Plug; Bard Davol Inc., NJ, USA) [10]. First, radical prostatectomy was performed with extended pelvic lymphadenectomy in a lower abdominal longitudinal incision. The protruded bladder walls were smoothly replaced into their original positions. The operative field was sterilized a second time and covered with an antibiotic film. Second, bilateral inguinal hernia repairs were performed using bilateral inguinal incisions that extended medially to the pubis (Figures 2(a) and 2(b)). The surgery time was 434 minutes. His postoperative course was uneventful. The patient ambulated and ingested food on operative day 2. On postoperative day 7, Cystogram revealed no leakage or relapse of IBHs. Thereafter, bladder catheter was removed and intravenous 2 g cefotiam dihydrochloride twice daily was discontinued. Eventually, no relapse of IBHs or PCa occurred.

### 2.2. Case 2

**Figure 3:**
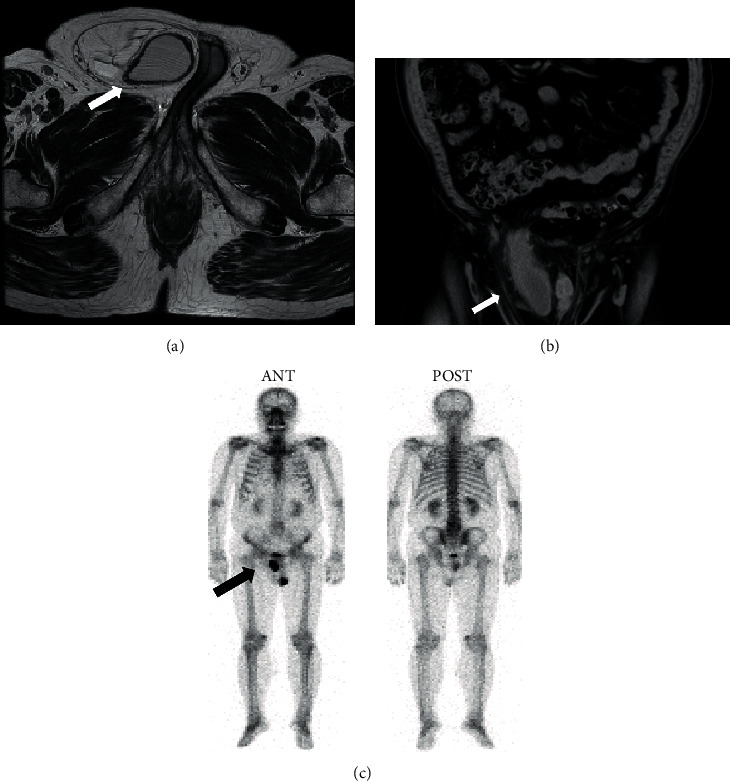
Images of case 2 showing the bladder in the right inguinal hernia. (a) Axial MRI image: the arrow indicates the IBH. (b) Coronal CT image: the arrow indicates the IBH. (c) BS image: the arrow indicates the IBH. BS: bone scintigraphy; MRI: magnetic resonance imaging; CT: computed tomography; IBH: inguinal bladder hernia.

A 75-year-old man with nocturia and urge incontinence was referred to the Department of Urology, Atsugi City Hospital, Japan. He experienced right inguinal painless swelling that gradually increased in size for 30 years. His medical history was unremarkable. A right scrotal mass was detected (20 cm in diameter). The mass was soft, non-transilluminated, and nonreducible without bowel sounds. His body mass index was 29.43 kg/m^2^, and PSA level was 4.17 ng/mL. PCa was diagnosed with a transrectal prostate needle biopsy, and the Gleason score was 3 + 4. His CT, magnetic resonance imaging (MRI), and bone scintigraphy (BS) demonstrated a right IBH without metastasis (Figures 3(a)–3(c)). He was diagnosed with intermediate-risk PCa accompanied by the right herniation of the bladder. Simultaneous IBH repair and RRP were performed. During the operation, the bladder was squeezed out of the expanded inguinal neck (from 2 cm to 4 cm in diameter) and placed in its original position using a lower abdominal longitudinal incision. Subsequently, radical prostatectomy with pelvic extended lymph node dissection was performed. Finally, a right inguinal incision was made. The enlarged inguinal ring was repaired using a polypropylene mesh (Lichtenstein technique, PerFix Light Plug; Bard Davol Inc., NJ, USA) after concealment with the endopelvic fascia [9]. The operative time was 243 minutes. The patient ambulated and ingested food the next postoperative day. He received intravenously 2 g cefotiam dihydrochloride twice daily until the first postoperative Cystogram. The bladder catheter was removed after this postoperative normal Cystogram on postoperative day 7. The patient's postoperative course was uneventful, and the histopathological findings of the prostate revealed adenocarcinoma. Three months later, neither IBH relapse nor PCa recurrence was observed.

## 3. Discussion

IBH is defined as an acquired direct inguinal hernia with the bladder pulled into the hernia, together with a sheath of the peritoneum, which forms its sac. It can be classified into 3 types according to their relationships with the peritoneum [2]. Type І is a paraperitoneal hernia, with the extraperitoneal portion of the hernia lying along the medial wall of the sac. This is the most frequently observed IBH type, and it was noted in both cases. Type II is an intraperitoneal hernia, with the bladder completely covered by the peritoneum in the hernia sac. Type III is an extraperitoneal hernia, with the peritoneum remaining in the abdomen and the bladder herniating. Different factors can be associated with the pathophysiology of IBHs that include (i) bladder outlet obstruction (e.g., benign prostatic hyperplasia and bladder neck or urethral strictures), (ii) obesity, and (iii) decreased bladder tone and weakness of the pelvic musculature, which occur in an aging population [2, 4, 11, 12]. In case 2, we hypothesized that the bladder wall gradually slid into the scrotal canal due to the progression of obesity and lower urinary tract symptoms (LUTs). However, the mechanism for IBH occurrence was unclear in case 1.

As small IBHs are generally asymptomatic, preoperative diagnosis of IBHs is difficult, and such hernias are commonly diagnosed incidentally during surgery. Nevertheless, it is important for surgeons to be aware of IBHs because bladder injury during herniorrhaphy can unknowingly lead to infection, sepsis, or even death [12]. In contrast, large IBHs have the classical presence of LUTs such as two-stage micturition, frequency, urgency, nocturia, dysuria, and hematuria [6].

Oruc et al. reviewed a total of 116 patients with IBH in 2004 [12]. In their review, 13 cases (11.1%) showed malignancy. Nine of those cases had bladder carcinoma, three cases had prostate carcinoma, and one case was reported to have a neoplasm. An additional 51 IBH cases that underwent surgical procedures were evaluated after their review [4, 8, 11, 13–47]. In our comprehensive review, which included the two current cases, five cases showed concomitant PCa. One of these cases involved a Russian patient, and there was no detailed treatment information; two other cases were treated with hormone therapy for PCa [5]. To the best of our knowledge, this is the first report on the simultaneous repair of IBHs and RRPs.

In the review of 51 cases (Table 1), the mean ± SD patient age was 60.6 ± 12.3 years and there was only 1 female case. Most cases underwent repair of the IBH with an inguinal incision or an extended medial incision (77.5%). Only 6 cases underwent minimally invasive surgery (1 robot-assisted procedure and 5 laparoscopic procedures). In approximately three-fourths of the reviewed cases, a mesh was used for IBH repair. RRP is classified as a clean-contaminated operation according to the Centers for Disease Control and Prevention (CDC) because of contamination of the surgical field by urine [48]. In herniorrhaphy, incisions were classified from the lower midline incision for RRP, and polypropylene mesh was concealed from clean-contaminated wound. Choi et al. and Manoharan et al. mentioned that simultaneous repair of inguinal hernias during RRP was effective and technically feasible regardless of mesh usage. Similarly, two surgical options were indicated. The first was to make an additional inguinal incision and perform a classic hernioplasty. The second was to perform a preperitoneal hernioplasty with an incision. The possibility of complications related to urinary contamination was lower with the first option. However, the disadvantages associated with the first approach are a high likelihood of wound infection, increased discomfort and wound pain, long recovery time, increased operative time, and additional use of anesthesia [49, 50]. In these cases, the first option was selected because it strictly kept wounds separated and sterilized according to the preoperative design.

The bladder was resected in 18.0% of cases that underwent IBH surgery. Bisharat et al. suggested that bladder resection was indicated if a diverticulum, incidental tumor, or necrosis was present. In addition, it was also indicated for preventing bladder strictures that can occur if hernia neck was less than 0.5 cm in diameter [4]. In this case report, hernia necks were over 0.5 cm in diameter (approximately 3.0 cm and 2.0 cm in case 1 and case 2, respectively). No indications for bladder necrosis or ischemia were observed. Therefore, the bladders were entirely preserved by compressing them out through the expanded hernia rings.

The advantages of this approach involving simultaneous treatment of IBHs and localized PCa compared with the two-step procedure include the following: (i) compressing the bladder through the pelvis was more protective, smooth, and forceful than in groin incision; (ii) it allowed for an easy observation of the compressed bladder in the pelvis; (iii) swift actions were possible if it was necessary to resect bladder partially; and (iv) it prevented recurrence of IBHs during elective surgery.

## 4. Conclusions

We successfully performed a simultaneous treatment of IBHs and localized PCa through a safe repair of IBHs (using mesh) and RRPs. Supposedly, this simultaneous repair of IBHs and RRP with dual incisions is suitable.

## Figures and Tables

**Table 1:** Demographics of 51 patients with inguinal bladder hernia.

Age (ys. mean ± SD, range)	60.6 ± 12.3 (30-79)
Gender (%)	
Male	50 (98.0)
Female	1 (2.0)
Type of surgical incision (*n* = 40) (%)	
Single	31 (77.5)
Dual	3 (7.5)
Robot-assisted	1 (2.5)
Laparoscopic	5 (12.5)
Mesh implant (*n* = 39) (%)	
Use	29 (74.4)
Nonuse	10 (25.6)
Resection of the bladder (%)	
Yes	9 (17.6)
No	43 (82.4)

## Data Availability

The datasets generated and/or analyzed during the current study are available from the corresponding authors on reasonable request, but no information infringing on the privacy of the participants will be given.
